# Coastal sharks supply the global shark fin trade

**DOI:** 10.1098/rsbl.2020.0609

**Published:** 2020-10-28

**Authors:** Kyle S. Van Houtan, Tyler O. Gagné, Gabriel Reygondeau, Kisei R. Tanaka, Stephen R. Palumbi, Salvador J. Jorgensen

**Affiliations:** 1 Monterey Bay Aquarium, Monterey, CA 93940, USA; 2 Nicholas School of the Environment, Duke University, Durham, NC 27708, USA; 3 Changing Ocean Research Unit, Institute for the Oceans and Fisheries, University of British Columbia, Vancouver, BC, Canada; 4 Department of Ecology and Evolutionary, Yale University, New Haven, CT 06520, USA; 5 Department of Biology, Hopkins Marine Station, Stanford University, Pacific Grove, CA 93950, USA

**Keywords:** wildlife trafficking, IUU fishing, elasmobranchs, barcoding, spatial species distribution models

## Abstract

Progress in global shark conservation has been limited by constraints to understanding the species composition and geographic origins of the shark fin trade. Previous assessments that relied on earlier genetic techniques and official trade records focused on abundant pelagic species traded between Europe and Asia. Here, we combine recent advances in DNA barcoding and species distribution modelling to identify the species and source the geographic origin of fins sold at market. Derived models of species environmental niches indicated that shark fishing effort is concentrated within Exclusive Economic Zones, mostly in coastal Australia, Indonesia, the United States, Brazil, Mexico and Japan. By coupling two distinct tools, barcoding and niche modelling, our results provide new insights for monitoring and enforcement. They suggest stronger local controls of coastal fishing may help regulate the unsustainable global trade in shark fins.

## 1. Introduction

**Figure 1.**
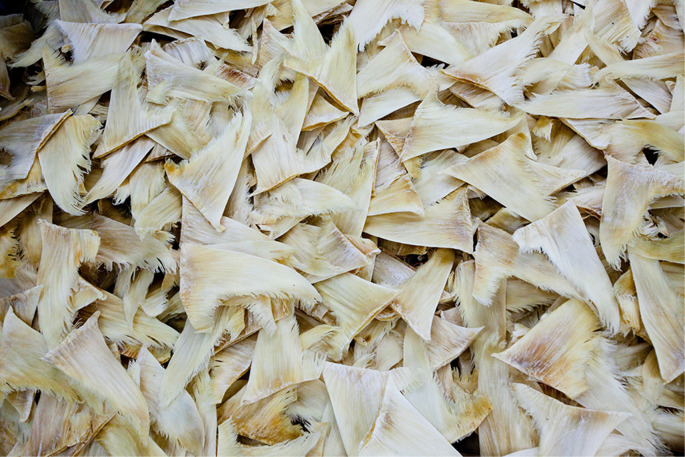
Market collections of shark fins are not easily identified to species. DNA barcoding techniques are revealing a greater number of threatened and coastal sharks from stockpiles of intact shark fins, processed fins (pictured) and fin products. Image credit: Paul Hilton, used with permission.

Scientists have long sought diagnostic tools to improve monitoring biodiversity in wild ecosystems and in markets [1,2] at a scale that matches their occurrence and exploitation [3,4]. Such tools could be vital for assessing the wildlife trade, where the species and geographic origin are often difficult to diagnose from traded products [5]. For shark fins alone, the trade is valued at nearly US$ 400 million and kills perhaps 100 million sharks annually [6,7]. Efforts to monitor the shark fin trade, however, have been impeded in part by extensive processing of the marketed products (figure 1). Though some fins are traded intact, many are traded with the skin and other morphological features removed, and mixed in stockpiles, defying easy cataloguing.

Advances in genetic sequencing, marker selection and global sequence libraries have increased the output and lowered the cost of diagnostic species identification of wildlife products. Separately, improvements in species distribution modelling [8–10] now allow probabilistic mapping of species occurrence from derived environmental niches. Such niche models have been successfully applied to marine fisheries, for example, to reduce bycatch and predict longline fishing effort (e.g. [11,12]). For the shark fin trade, coupling the two approaches into a single analysis may help narrow a broad problem by describing the most probable shark fishing locations, potentially revealing geographic, which will improve monitoring and conservation.

PCR-based sequencing of individual fins provided the first species key to interpret trade records and estimate species composition. One pioneering analysis [3] of fins from the Hong Kong auction, which accounts for half of the global fin trade, revealed the dominance of pelagic sharks (*Prionace glauca*, *Carcharhinus longimanus*, *Isurus* spp. and *Alopias* spp.) and focused management on high-seas fishing. However, the method was difficult to scale up and provided little information on rare species. A DNA barcoding approach using BOLD and BLAST taxonomy databases [13] with extensive sequence libraries provides an improved ability to identify larger numbers of species. Using this approach, four recent studies [13–16] revealed the source species of over 5000 individual shark fins from markets in Hong Kong, Vancouver, San Francisco and northern Brazil. These studies provide a novel influx of robust information, identifying a wide variety of threatened species, and marking a new and increased ability to identify processed market samples to the species level.

Here, we combine these existing barcoding data with species distribution models (SDMs) to understand the geographic sources of traded shark fins. This generates a probability surface of the global ocean that describes the likely locations fished to produce the market fins. Finally, we assess the relative importance of areas inside and outside Exclusive Economic Zones (EEZs) and accumulate and rank each nation's contribution. Our results revise the estimates from 20 years ago, suggesting that the greatest opportunities for regulating the shark fin trade are in coastal regions.

## 2. Methods

Published studies [13–16] used cytochrome oxidase (COI) sequencing to provide robust species identifications for 5327 shark fins sampled at markets in Brazil, Canada, China and the United States (electronic supplementary material, table S1 and figure S1). Previous analyses generated SDMs for 51 of these species from open-access occurrence data at Global Biodiversity Information Facility, FishBase and Ocean Biodiversity Information System. Each SDM is the ensemble of four environmental niche models (BioClim, Boosted Regression Trees, Maxent and Artificial Neural Networks [8]) and displays the probability of occurrence globally at a 0.5° × 0.5° resolution. To account for potential sampling biases in the source data, we conducted randomized cross-validations for all SDMs and evaluated model performance (see electronic supplementary material, table S2). AquaMaps [17] provided SDM outputs for eight additional species (see electronic supplementary material, table S1) using similar methods.

We simulated probability maps of shark fishing by integrating the barcoding results and SDMs. For each market study, *φ_i_* is the compositional proportion (*φ*) for each identified species (*i*). With each SDM raster, we conducted *φ_i_* × 10^6^ Bernoulli trials in R [18] where the *dplyr* package [19] ‘sample_n' function randomly selected pixels and the R ‘binom' function queried them (1 = presence, 0 = absence). This routine adds stochasticity and simulates fishery captures by reducing continuous probability in the SDM surface to discrete events. We summed all SDM trials for each barcoding study and rescaled the accumulated surfaces from 0 to 1 for standardized comparison. Next we mapped results, summed and compared probability densities within and outside EEZs, and calculated and ranked the total contributions from each sovereign nation. All analyses and visualizations were conducted in the R environment. All scripts and data are provided open access (https://osf.io/xvrmk/, [20]).

## 3. Results and discussion

Our analyses suggest most harvested shark fins originate within EEZs rather than in high-seas regions, counter to previous assessments [3]. Abundant and widespread species such as blue sharks (*P. glauca*) remain the dominant species in fins from the Hong Kong market hub [15]. However, even there, genetic barcoding revealed an additional 40 range-restricted coastal species (electronic supplementary material, figures S1 and S2), while studies from other markets [13,14,16] show a greater proportion of coastal sharks.

**Figure 2.**
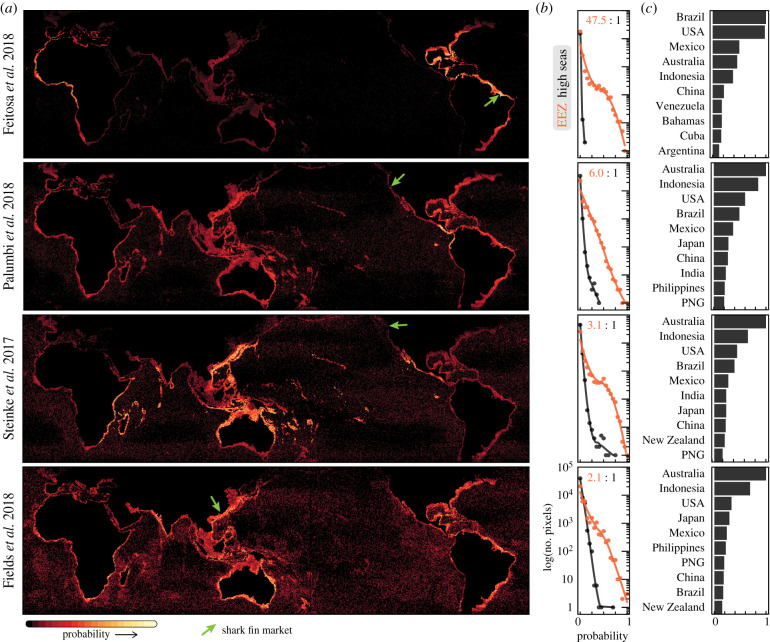
Global origins of shark fins. Probability of origin for shark fins sampled from markets (*a*) across the global ocean, (*b*) by EEZ jurisdiction and (*c*) ranked by top sovereign nation contributors. Maps accumulate probability from each species' niche model values, where each species is proportionately rated by the number of fin identifications in four studies [13–16]. Most activity occurs near-shore, within EEZs, and the reported ratio in (*b*) is the relative probability. (*c*) Australia, Indonesia, the United States, Brazil, Mexico and Japan (see electronic supplementary material, table S3) represent the nations contributing the most shark fins to the global market. PNG, Papua New Guinea.

Figure 2*a* plots the probability of occurrence for all species, according to fin identifications in four market studies. The similar geographic pattern of the Hong Kong, Vancouver and San Francisco market sources [13,15,16] reflects that China is the established aggregating node that receives, processes and supplies a majority of fins to global markets [16]. The Brazil-based study [14] sampled fins in a series of local wet markets and indicates a dominance of fishing activity in coastal Brazil and the Caribbean (figure 2*a,c*). Unsurprisingly, the relative frequency of modelled shark fishing within EEZs to the high seas (figure 2*b*) is greatest in the Brazil market (48 : 1). Yet even in the recent Hong Kong study, shark fins originated within EEZs by a 2 : 1 ratio.

Since the largest portion of shark harvest originates within EEZs, there is an urgent need for nations to adopt enforceable conservation measures within their jurisdictions. The findings also suggest that progress is not limited to international negotiations over high-seas fishing and can also be targeted to EEZ regions with more jurisdictional oversight. Figure 2*b* shows that probability of origin declines steeply with frequency (note the log-scaled *y*-axis), as the niche models have focused the likely locations of shark fishing to coastal zones (figure 2*a*). Small-scale vessels operating in coastal waters, however, present multiple complications for monitoring, including enforcement gaps, stockpiling and transhipment [5]. Progress on these persistent challenges within the EEZs of Australia, Indonesia, the United States, Brazil, Mexico and Japan (figure 2*c*; electronic supplementary material, table S3) may be especially effective. These results differ starkly from previous summaries of the shark fin trade that relied on official customs statistics [7]. Such records are widely considered unreliable, double count re-export data, and do not represent the geographic origin where shark harvests occurred [7]. Our approach helps to resolve these issues.

Despite some geographic differences in the sourcing regions of the four markets, the barcoding results consistently revealed a high number of threatened and rare species. Averaged across studies, 52% of species identified (range 47–60%), and 61% of fins (range 35–75%) came from species classed as either threatened (CR, EN or VU) or ‘data deficient' (DD) by the IUCN (see electronic supplementary material, table S1). Some species are further protected by the Convention on the International Trade in Endangered Species and various national policies. Previously, genetic analyses of the global composition of shark species were limited to the detection of the most common species that were also morphologically identifiable [3]. That half of traded fins from recent barcoding studies are from species of conservation concern reveals a pressing need for increased monitoring, management and enforcement of the shark fin trade.

Some shark fisheries are considered sustainable. The spiny dogfish (*Squalus acanthias*) for example has been suggested as a model shark fishery [21] and potentially could supply shark fin markets. However, S. *acanthias* is harvested for meat and does not produce marketable fins, and there existed zero S. *acanthias* identifications in the barcoding analyses we compiled. Other reportedly sustainable shark fisheries [21] represent just a small minority of fin identifications from markets (electronic supplementary material, table S1). Sustainable choices to supply markets and consumer demand for shark fins may therefore remain elusive, furthering support for strong controls on the shark fin trade [22,23].

We attribute the identification of more species and rare species to growth in barcoding libraries and attribute the coastal concentration of shark fishing (figure 2) to this influx of species and the niche-based SDMs. By proportionally weighting suitable habitats, the SDMs provide a more informed assessment of where encountering those species and their harvests most likely occurs [11]. However, as the chronic exploitation of pelagic sharks in high-seas fisheries has collapsed many shark populations [24], our results here may also reflect a serial shift from distant fleets to near-shore fishing. In addition, expanded sampling beyond Hong Kong retailers may have revealed previously undetected regional differences in supply chains. Either way, this raises new concerns as near-shore shark populations have also seen dramatic declines [25], are typically less abundant, have smaller geographic distributions and often have less management [15].

## Supplementary Material

A fuller description of the results and more transparency on the modeling process and results

## Data accessibility

All data and code for this study are available at a third-party open-access repository (https://osf.io/xvrmk/) [20].

## References

[1] Janzen DH 2004 Now is the time. Phil. Trans. R. Soc. Lond. B 359, 731–732. (10.1098/rstb.2003.1444) 15253359 PMC1693358

[2] DeSalle R, Egan MG, Siddall M 2005 The unholy trinity: taxonomy, species delimitation and DNA barcoding. Phil. Trans. R. Soc. B 360, 1905–1916. (10.1098/rstb.2005.1722) 16214748 PMC1609226

[3] Clarke SC, Magnussen JE, Abercrombie DL, Mcallister MK, Shivji MS 2006 Identification of shark species composition and proportion in the Hong Kong shark fin market based on molecular genetics and trade records. Conserv. Biol. 20, 201–211. (10.1111/j.1523-1739.2005.00247.x) 16909673

[4] Wasser SK, Clark WJ, Drori O, Kisamo ES, Mailand C, Mutayoba B, Stephens M. 2008 Combating the illegal trade in African elephant ivory with DNA forensics. Conserv. Biol. 22, 1065–1071. (10.1111/j.1523-1739.2008.01012.x) 18786100

[5] Miller EA, McClenachan L, Uni Y, Phocas G, Hagemann ME, Van Houtan KS 2019 The historical development of complex global trafficking networks for marine wildlife. Sci. Adv. 5, eaav5948 (10.1126/sciadv.aav5948) 30957017 PMC6449156

[6] Worm B, Davis B, Kettemer L, Ward-Paige CA, Chapman D, Heithaus MR, Kessel ST, Gruber SH 2013 Global catches, exploitation rates, and rebuilding options for sharks. Mar. Pol. 40, 194–204. (10.1016/j.marpol.2012.12.034)

[7] Dent F, Clarke S 2015 State of the global market for shark products. *FAO Fish. Aquacult. Tech. Pap*., no. 590. See http://www.fao.org/3/a-i4795e.pdf.

[8] Gagné TO, Reygondeau G, Jenkins CN, Sexton JO, Bograd SJ, Hazen EL, Van Houtan KS 2020 Towards a global understanding of the drivers of marine and terrestrial biodiversity. PLoS ONE 15, e0228065 (10.1371/journal.pone.0228065) 32023269 PMC7001915

[9] Reygondeau G 2019 Current and future biogeography of exploited marine exploited groups under climate change. In Predicting future oceans (eds Cisneros-Montemayor AM, Cheung WWL, Ota Y), pp. 87–101. Amsterdam, The Netherlands: Elsevier.

[10] Rogers AD et al. 2020 Critical habitats and biodiversity: inventory, thresholds and governance. Washington, DC: World Resources Institute.

[11] Crespo GO, Dunn DC, Reygondeau G, Boerder K, Worm B, Cheung W, Tittensor DP, Halpin PN 2018 The environmental niche of the global high seas pelagic longline fleet. Sci. Adv. 4, eaat3681 (10.1126/sciadv.aat3681) 30101192 PMC6082651

[12] Hazen EL et al. 2018 A dynamic ocean management tool to reduce bycatch and support sustainable fisheries. Sci. Adv. 4, eaar3001 (10.1126/sciadv.aar3001) 29854945 PMC5976278

[13] Palumbi S, Van Houtan K, Robinson K, Jorgensen S 2018 DNA analysis of a large collection of shark fins from a US retail shop: species composition, global extent of trade and conservation. *bioRxiv*, 433847 (10.1101/433847)

[14] Feitosa LM et al 2018 DNA-based identification reveals illegal trade of threatened shark species in a global elasmobranch conservation hotspot. Scient. Rep. 8, 3347 (10.1038/s41598-018-21683-5) PMC5820252 29463851

[15] Fields AT, Fischer GA, Shea SKH, Zhang H, Abercrombie DL, Feldheim KA, Babcock EA, Chapman DD 2018 Species composition of the international shark fin trade assessed through a retail-market survey in Hong Kong. Conserv. Biol. 32, 376–389. (10.1111/cobi.13043) 29077226

[16] Steinke D, Bernard AM, Horn RL, Hilton P, Hanner R, Shivji MS 2017 DNA analysis of traded shark fins and mobulid gill plates reveals a high proportion of species of conservation concern. Scient. Rep. 7, 9505 (10.1038/s41598-017-10123-5) PMC5573315 28842669

[17] Kaschner K et al 2019 AquaMaps: predicted range maps for aquatic species. See www.aquamaps.org, v 10/2019.

[18] R Core Team. 2018 R: a language and environment for statistical computing. Vienna, Austria: R Foundation for Statistical Computing See https://www.R-project.org.

[19] Wickham H et al. 2019 Welcome to the Tidyverse. J. Open Source Softw. 4, 1686 (10.21105/joss.01686)

[20] Van Houtan KS, Gagne T, Tanaka KR, Jorgensen SJ. 2020 Data and code from: Coastal sharks supply the global shark fin trade *Open Science Framework*. (10.17605/OSF.IO/XVRMK) PMC7655481 33108982

[21] Simpfendorfer CA, Dulvy NK 2017 Bright spots of sustainable shark fishing. Curr. Biol. 27, R97–R98. (10.1016/j.cub.2016.12.017) 28171764

[22] Sadovy de Mitcheson Y, Andersson AA, Hofford A, Law CSW, Hau LCY, Pauly D 2018 Out of control means off the menu. Mar. Pol. 98, 115–120. (10.1016/j.marpol.2018.08.012)

[23] Ferretti F et al 2020 Shark fin trade bans and sustainable shark fisheries. Conserv. Lett. 13, e12708 (10.1111/conl.12708)

[24] Baum JK, Myers RA, Kehler DG, Worm B, Harley SJ, Doherty PA 2003 Collapse and conservation of shark populations in the Northwest Atlantic. Science 299, 389–392. (10.1126/science.1079777) 12532016

[25] Martin SL, Van Houtan KS, Todd Jones T, Aguon CF, Gutierrez JT, Brent Tibbatts R, Wusstig SB, Bass JD 2016 Five decades of marine megafauna surveys from Micronesia. Front. Mar. Sci. 2, 116 (10.3389/fmars.2015.00116)

