# Supplementary material for: Coastal sharks supply the global shark fin trade
Source: Biol Lett. 2020 Oct 28;16(10):20200609. doi: 10.1098/rsbl.2020.0609 (PMC7655481; doi:10.1098/rsbl.2020.0609)
Supplement: A fuller description of the results and more transparency on the modeling process and results [file rsbl20200609supp1.docx]

**SUPPLEMENTARY ONLINE MATERIALS ACCOMPANYING THE ARTICLE:**

**“**Coupling DNA metabarcoding and species distribution models to source shark fins”

Kyle S. Van Houtan ([kvanhoutan@mbayaq.org](mailto:kvanhoutan@mbayaq.org)), Tyler O. Gagné ([engagrelyt@gmail.com](mailto:engagrelyt@gmail.com)), Gabriel Reygondeau ([gabriel.reygondeau@gmail.com](mailto:gabriel.reygondeau@gmail.com)), Kisei R. Tanaka ([ktanaka@mbayaq.org](mailto:ktanaka@mbayaq.org)), Salvador J. Jorgensen ([salvador.jorgensen@gmail.com](mailto:salvador.jorgensen@gmail.com)), and Stephen R. Palumbi ([spalumbi@stanford.edu](mailto:spalumbi@stanford.edu))

**This file includes:**

- **Further details on the Methods**
- **Table S1: Metadata of species identifications, threatened status, and distribution model sources.**
- **Figure S1. Species composition from four recent barcoding analyses of shark fins at market.**
- **Figure S2. Global probabilities of occurrence for 57 shark species derived from species distribution models.**
- **Table S2. Species Distribution Model output and performance statistics.**
- **Table S3. Top 40 nations modeled contributions to the shark fin trade.**

**Further details on the Methods**

This study uses the species occurrence data from previously published studies on marine biodiversity [1-3] to develop a standardized observation record for shark species to feed species distribution models (SDMs). These studies searched for occurrence records for shark species identified from barcoding analyses of fin market samples [4-7] in the Ocean Biogeographic Information System (OBIS) [8]; the Global Biodiversity Information Facility (GBIF) [9]; and Fishbase [10] databases.

As described previously [1, 3], a number of quality-control routines were performed to screen and filter the raw occurrence data. Briefly, all records without spatial information or that were not identified to the species level were removed and duplicate records reported in multiple databases were expunged and merged into a single record. This results in 805,235 unique and filtered observations for 51 shark species (see Table S2). Three species with fewer than 10 observation records (*Lamiopsis temminckii*, *Carcharhinus leiodon*, *Squalus brevirostri*) represent only 0.13% (7 of 5,327) of the cumulative fins identified (Table S1), and we do not expect their few observations to influence the sourcing maps we produce (Figure 2a). For an additional 8 species we use the SDM outputs from Aquamaps [11].

For each species, 15-fold 70/30 cross validations were run on the SDMs [12]. This process randomly splits the source data, where 70% of observations are set aside for model calibration and 30% of observations are assigned for model validation. Each iteration of this process—or each time this is done (“fold”)—we compute the area under the curve (“AUC”) of the receiver operating characteristic [13]. This process is repeated 15 times and the resulting AUC value is the average from each of the 15 folds. The AUC values for all shark species SDMS is greater than 0.80 (see Table S2). Aquamaps provides the remaining SDM outputs.

**Table S1: Metadata of species identifications, threatened status, and distribution model sources.** “Taxon”, “Family”, “Genus”, “Species”, and “Common Name” follow the previously reported format [6]. “IUCN” is the extinction risk (LC Least Concern, DD Data Deficient, NT Near Threatened, VU Vulnerable, and EN Endangered). “CITES” is the Appendix listing constraining international trade. “Range Model” is the published data source for the species distribution model [3, 11]. “Feitosa”, “Palumbi”, “Steinke”, and “Fields” are the first author of the published studies [4-7], numeric value reports the number of fin identifications, and “—” means no results reported. These data are also available as a CSV file at a third-party open access repository (<https://osf.io/xvrmk/>).


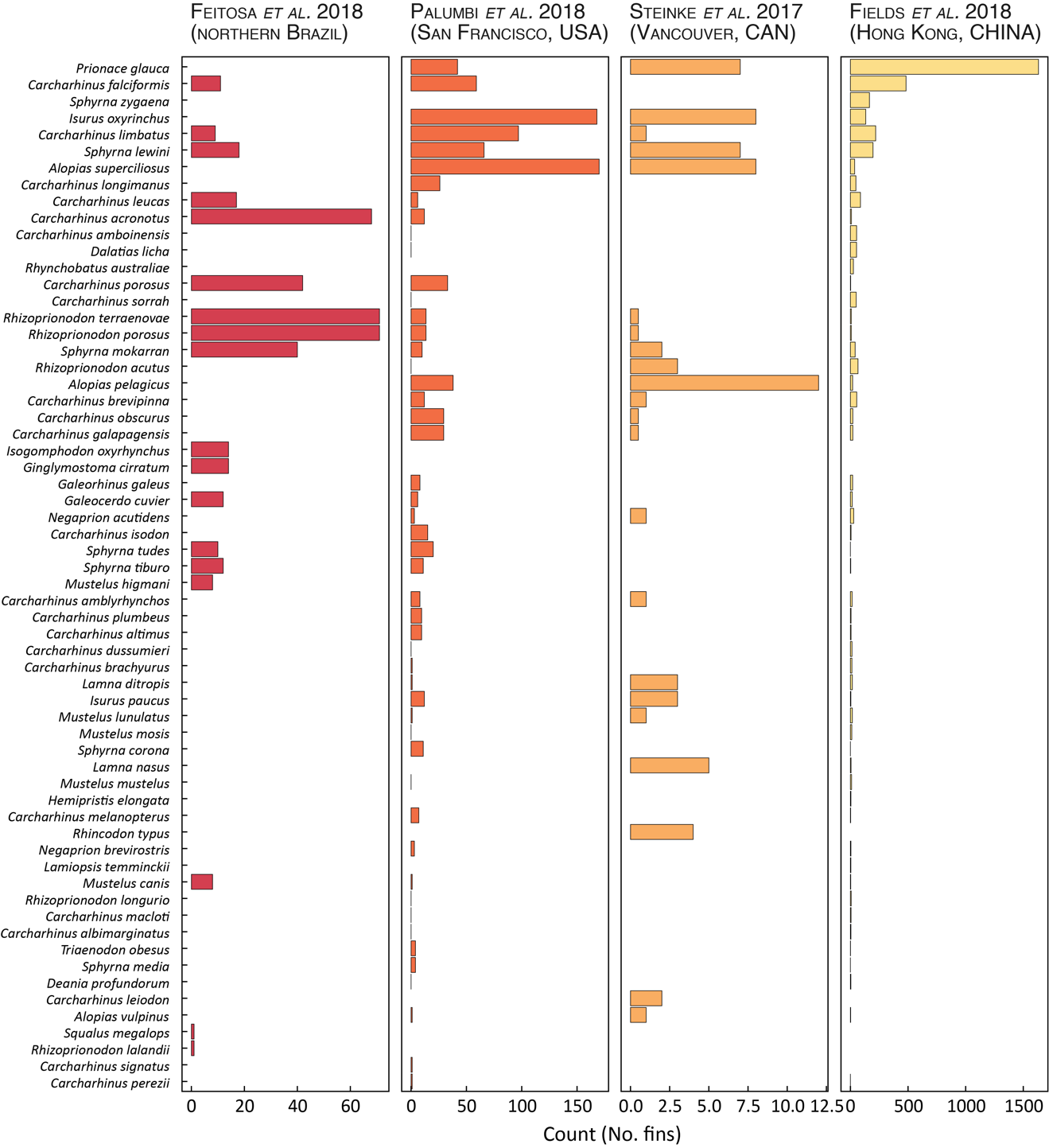


**Figure S1. Species composition from four recent barcoding analyses of shark fins at market.** DNA metabarcoding identifications of shark fins samples at point of sale markets in Fields et al. from Hong Kong, China [5]; in Steinke et al. from Vancouver, Canada [7]; in Palumbi et al. from San Francisco, USA [6]; and in Feitosa et al. from northern Brazil [4]. Identifications are reported in each study for each fin sampled and are standardized across studies using Cytochrome oxidase (COI) sequencing methods. These data underpin the market surveys used in our main text analyses (Figure 1).


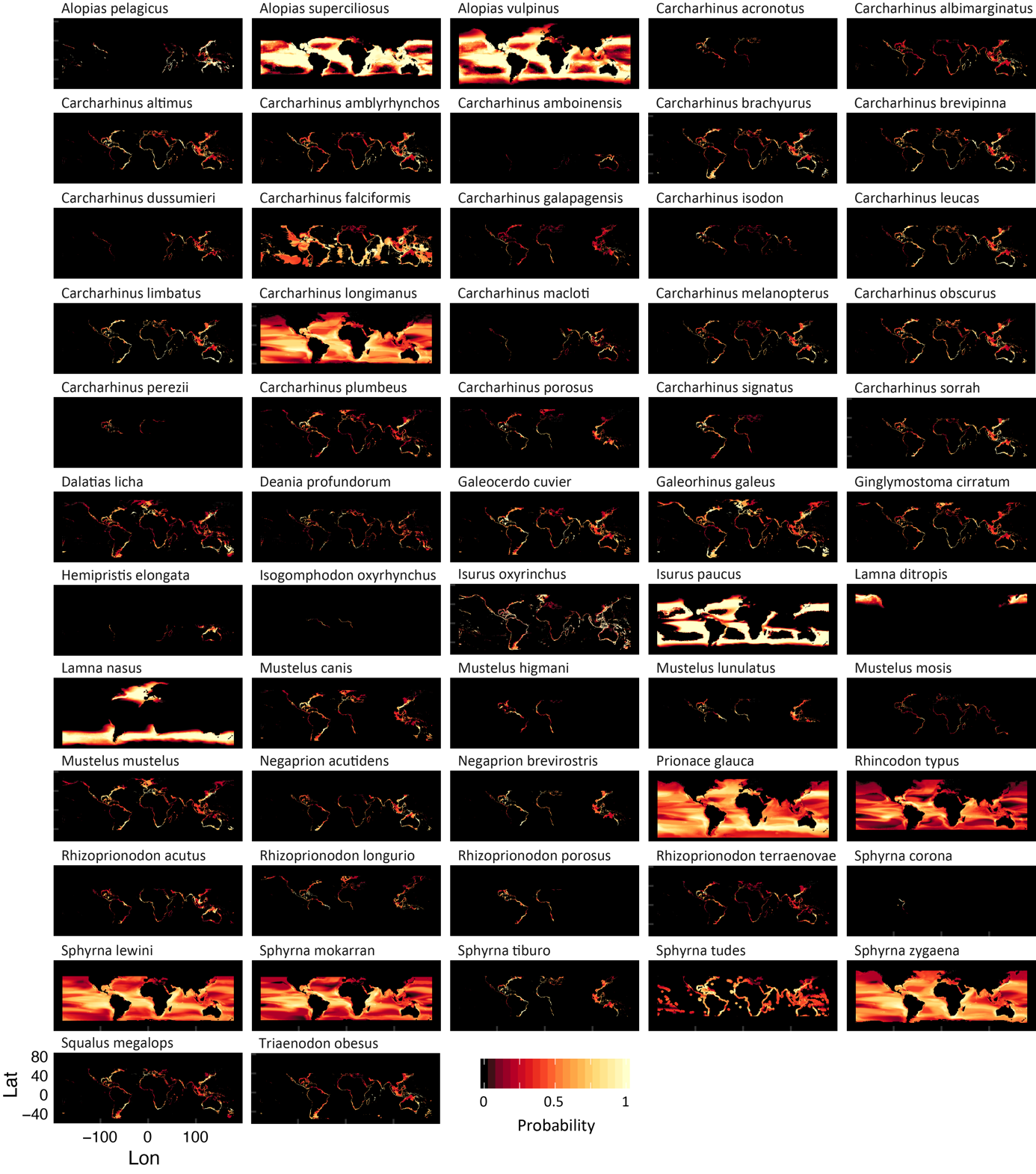


**Figure S2. Global probabilities of occurrence for 57 shark species derived from species distribution models.** Distributions are multivariate environmental niche models derived from accompanying factors cooccurring at species observation records. Probabilities are rescaled 0-1 where 0 is absence and 1 is the optimal niche. All species distributions are from the same methods [3], save 7 species (*Alopias* spp*, Isurus* spp, *Lamna ditropis*, *Lamna nasus*, and *Sphyrna corona*) whose data were inaccessible and derived elsewhere [11].

**Table S2. Species Distribution Model output and performance statistics.** First three columns of this table share the form of Table S1. “Unique records” is the total unique observation records from GBIF, OBS and FishBase (replicates removed). “Cells occur” is the number of grid cells (global, 0.5°x0.5° resolution) that contain occurrence data, “Cells predict” is the number of grid cells in the SDM output that contain the species. “Niche area” is the total predicted species distribution (in km^2^) from the SDM, and “AUC” is the area under the curve of the receiver operating characteristic, that evaluates each SDM model performance. AquaMaps SDMs were extensively validated and evaluated previously [14] and we do not report their AUC here.

**Table S3. Top 40 nations modeled contributions to the shark fin trade.** “Country” is sovereign nation, “Feitosa”, “Palumbi”, “Steinke”, and “Fields” represent the first author of the published studies [4-7], and the column value is the summed probability values from the model results. “Average” is the average of these probabilities across studies, and “Rank” is the sorted order of “Average” from greatest to least.

**REFERENCES**

[1] Gagné, T.O., Reygondeau, G., Jenkins, C.N., et al. 2020 Towards a global understanding of the drivers of marine and terrestrial biodiversity. PLoS One 15, e0228065.

[2] Reygondeau, G. 2019 Current and future biogeography of exploited marine exploited groups under climate change. In Predicting Future Oceans (eds. A.M. Cisneros-Montemayor, W.W.L. Cheung & Y. Ota), pp. 87-101. Amsterdam, Elsevier.

[3] Rogers, A.D., Aburto-Oropeza, O., Appeltans, W., et al. 2020 Critical Habitats and Biodiversity: Inventory, Thresholds and Governance. Washington DC, World Resources Institute.

[4] Feitosa, L.M., Martins, A.P.B., Giarrizzo, T., et al. 2018 DNA-based identification reveals illegal trade of threatened shark species in a global elasmobranch conservation hotspot. Scientific Reports 8, 3347.

[5] Fields, A.T., Fischer, G.A., Shea, S.K., et al. 2018 Species composition of the international shark fin trade assessed through a retail‐market survey in Hong Kong. Conserv Biol 32, 376-389.

[6] Palumbi, S., Van Houtan, K., Robinson, K. & Jorgensen, S. 2018 DNA analysis of a large collection of shark fins from a US retail shop: species composition, global extent of trade and conservation. bioRxiv, 433847.

[7] Steinke, D., Bernard, A.M., Horn, R.L., et al. 2017 DNA analysis of traded shark fins and mobulid gill plates reveals a high proportion of species of conservation concern. Scientific reports 7, 9505.

[8] Grassle, J.F. 2000 The Ocean Biogeographic Information System (OBIS): an on-line, worldwide atlas for accessing, modeling and mapping marine biological data in a multidimensional geographic context. Oceanography 13, 5-7.

[9] GBIF_The_Global_Biodiversity_Information_Facility. 2020 What is GBIF? Available from <https://www.gbif.org/what-is-gbif>.

[10] Froese, R. & Pauly, D. 2010 FishBase. (Fisheries Centre, University of British Columbia.

[11] Kaschner, K., Kesner-Reyes, K., Garilao, C., et al. 2019 AquaMaps: Predicted range maps for aquatic species. <www.aquamaps.org>, v 10/2019.

[12] Guisan, A., Thuiller, W. & Zimmermann, N.E. 2017 Habitat suitability and distribution models: with applications in R, Cambridge University Press.

[13] Hernandez, P.A., Graham, C.H., Master, L.L. & Albert, D.L. 2006 The effect of sample size and species characteristics on performance of different species distribution modeling methods. Ecography 29, 773-785. (doi:10.1111/j.0906-7590.2006.04700.x).

[14] Ready, J., Kaschner, K., South, A.B., et al. 2010 Predicting the distributions of marine organisms at the global scale. Ecological Modelling 221, 467-478.
